# AUTONOMIC ACTIVITY AND THE AGING BRAIN

**DOI:** 10.1093/geroni/igad104.0215

**Published:** 2023-12-21

**Authors:** Mara Mather

## Abstract

Aging affects both autonomic activity and the brain regions that help modulate autonomic activity. In this symposium, we present new findings on how the relationships between autonomic activity and the brain change in aging. In addition, we demonstrate that modulating autonomic activity can affect the aging brain and emotional and cognitive functions controlled by the brain. Kathy Liu will present research from over 600 participants showing that, unlike younger adults who show the expected positive relationship between heart rate variability (HRV) and brain and behavioral indicators of emotion regulation, older adults showed a negative relationship between HRV and emotion regulation. Julian Thayer will present findings on how blood pressure and total peripheral resistance relate to brain structure. Richard Song will present functional MRI data revealing that the older brain shows less blood oxygen level dependent (BOLD) response to physiological fluctuations than younger brains. Jungwon Min will present findings that random assignment to daily biofeedback to either increase or decrease heart rate oscillations had a large effect on plasma amyloid-β. Mara Mather will present a new theoretical model positing that older brains attempt to compensate for hyperactive peripheral sympathetic activity, and that this ventromedial prefrontal compensatory activity leads to the biases in attention and memory known as the age-related positivity effect. Together, the empirical findings and theoretical perspectives presented in this symposium indicate that the autonomic system exerts important influences over the aging brain and that this provides a significant opportunity for intervening to improve brain health.

